# Importance of weightlifting performance analysis in anti-doping

**DOI:** 10.1371/journal.pone.0263398

**Published:** 2022-02-04

**Authors:** Hyunji Ryoo, Seok Ryu, Daejung Kim, Hayun Jeong, Denny Eun, Sang-Hoon Suh

**Affiliations:** 1 Department of Physical Education, Yonsei University, Seoul, Republic of Korea; 2 Department of Electrical and Electronic Engineering, Yonsei University, Seoul, Republic of Korea; 3 Korea Anti-doping Agency, Seoul, Republic of Korea; 4 Department of Nutritional Sciences, Faculty of Arts and Science, University of Toronto, Toronto, Canada; 5 Sport Science Laboratory, Shingu College, Seongnam, Republic of Korea; University of Lausanne, SWITZERLAND

## Abstract

We examined the potential roles of the athlete’s performance passport (APP) for doping detection by analyzing the relationship between weightlifting performance and sanction status. For the present study, performance data of ‘not-sanctioned’ (26740 datasets) and ‘sanctioned’ (289 datasets) male athletes were acquired from the website of the International Weightlifting Federation (www.iwf.net). One-way ANOVA, correlation analysis, and t-tests were used to analyze the relationship between athletes’ use of doping and their performances across age and body weight. Athletic performance was significantly greater for athletes in the sanctioned group than those of the same age group who were not sanctioned, and this performance difference between the two groups was the greatest in their late thirties at 20.6% (not-sanctioned 292.0kg vs. sanctioned 352.3kg) (*p* < 0.05). From the age group analysis, out of 289 sanctioned cases, 84 cases, which was the largest proportion, were found within the top 10–25% of their performances. When stratified by body weight, athletic performance was significantly greater for the sanctioned group than the not-sanctioned group, and this performance gap was the greatest in the bodyweight category of 96 at 18.6% (not-sanctioned 310.1kg vs. sanctioned 367.8kg) (*p* < 0.05). From the body weight category analysis, out of 289 sanctioned cases, 75 cases, which was the largest proportion, were found within the top 10–25% of their performances. Additionally, the mean difference in performance between not-sanctioned and sanctioned groups was the largest in the body weight category of 67kg in the ages of 15–19 at 20% (not-sanctioned 234.6kg vs. sanctioned 281.5kg). These results are interpreted to mean that in male weightlifters 1) sanctioned athletes were detected in all ranges of performances regardless of age and body weight, 2) there were even higher rates of sanctioned athletes who performed within the top 10–25% of each age group and body weight category, 3) there were significant differences in performance between not-sanctioned and sanctioned group for all body weight categories, excluding +109, in the ages of 15–19 and 20–24, 4) therefore, performance data can be effectively used to better target suspected athletes for doping testing.

## Introduction

The fight against doping has been a critical issue in sports and requires ongoing effort to ensure fairness. Fortunately, due to the valuable efforts that have been made, there is far greater awareness of doping in sports, as shown in the results of previous studies [1]. Since the 1960s, the fight against doping has developed considerably, from the detection of banned substances to the monitoring and analysis of indirect biomarkers in urine and blood, called the athlete’s biological passport (ABP). Since the introduction of the ABP, it has become a significant tool for anti-doping [2, 3], but there is an additional need to enhance effectiveness in anti-doping.

It is reasonable to use athletic performance data in order to improve the targeting of athletes in doping control since the main reason for doping is to improve athletic performance. This is based on Article 4.5.3.b in the world anti-doping code (WADC) international standard for testing and investigations, which states that “sport performance history, including in particular sudden major improvements in performance, and/or sustained high performance without a commensurate testing record” should be made the subject of target testing and is likely to indicate possible doping/increased risk of doping [4]. To identify unusual improvements in athletic performances possibly caused by doping, the so-called athlete’s performance passport (APP) requires longitudinal monitoring of them. In a recent study, Schumacher and Pottgeisser (2009) observed that middle- and long-distance running performances enhanced considerably after 1990, one year before which erythropoietin (EPO) became commercially available [5]. In addition, they observed a marked decline in performances in discus throws after introducing the out-of-competition test (OOCT) in 1988, suggesting the reduction of possible abuse of anabolic steroids. Results of that study were interpreted to indicate the link between athletic performances and possible doping, and increased the likelihood of tracking athletic performances in doping control. However, expected ranges in athletic performances due to the application of factors such as optimal conditioning, training, and nutrition, should be differentiated from disproportionate improvements.

To our knowledge, few studies have examined the application of athletic performance data in doping testing, and no studies have yet investigated the relationship between weightlifting performance and possible doping. Therefore, the purpose of this study was to comparatively analyze the athletic performance of male weightlifters based on their sanction status (sanctioned vs. not-sanctioned) to test the hypothesis that athletic performances can be used to detect possible doping. There have been significant improvements in anti-doping measures, but limitations still exist. Among those tested, positive test results provide definitive proof of doping, which results in sanctions. However, since not all athletes are tested, it may be erroneous to conclude that all athletes, who are not caught or tested, are clean. Our study focused specifically on determining performance differences between ‘sanctioned’ athletes who have been caught doping and ‘not-sanctioned’ athletes, which includes clean athletes, as well as those who doped, but were not caught. Since athletes with certain levels of performance can be subject to doping tests, our study examined whether analyzing the distribution can reveal insights for improving the efficiency of target testing. In addition to comparing the differences between sanctioned and not-sanctioned athletes, our analyses further investigated various factors, such as whether more of the top-performing athletes were caught or if performance played any significant role. Identifying the relationship between athletes’ performance and being sanctioned through enhanced methods of data retrieval and analyses can be a key piece of evidence in supporting the validity of the APP. With further research on the APP, there is much potential for developing an intelligence-led approach for better targeting athletes at high risk of doping, thereby further strengthening the effectiveness of doping tests and existing anti-doping systems.

## Materials and methods

**Fig 1:**
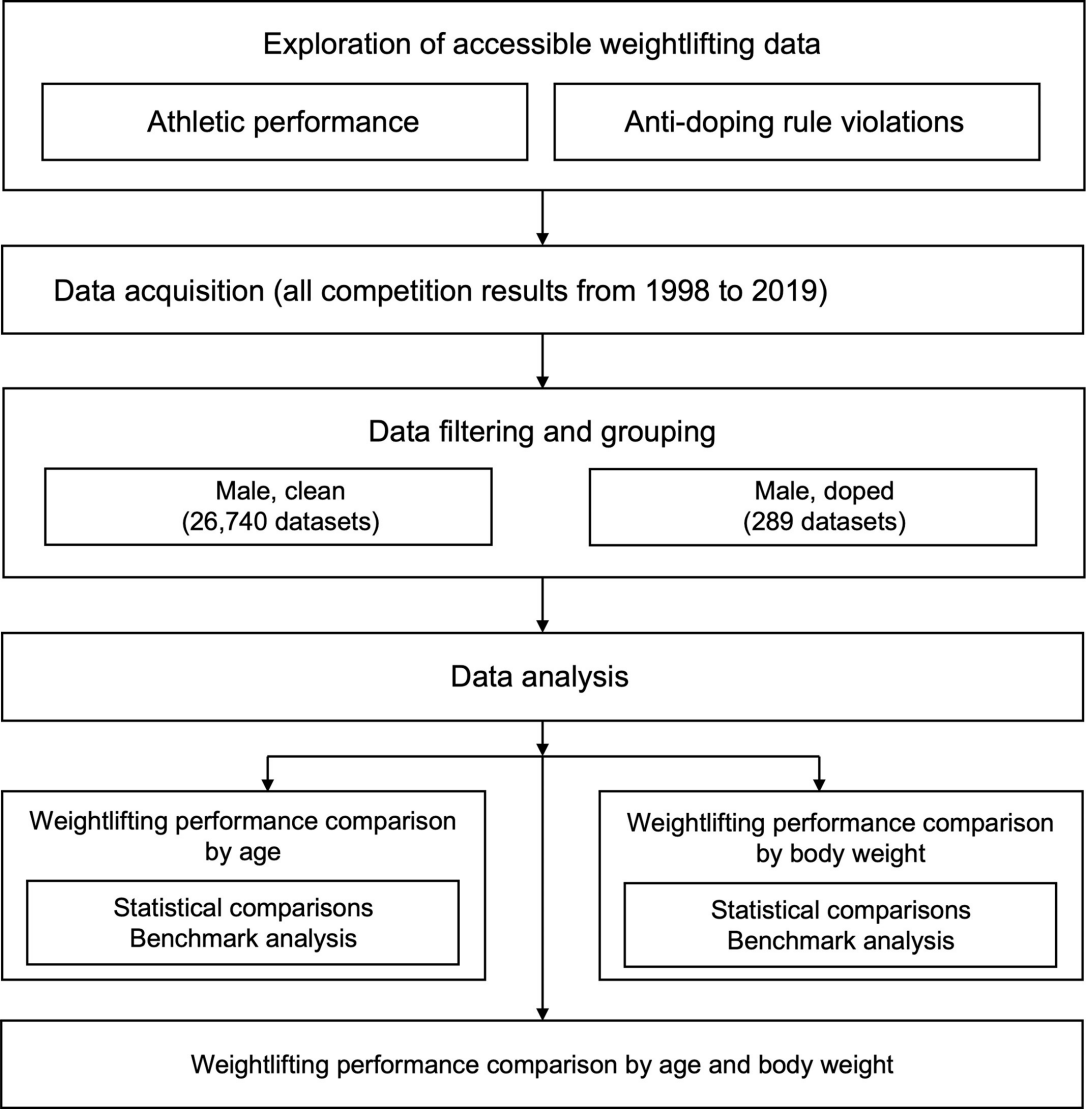
Experimental design of the study.

We analyzed male weightlifters’ performance data to establish the potential roles for applying the APP in anti-doping. Among the various sporting events, weightlifting was chosen for the present study as continuous performance data, such as total weight lifted in kilograms, can be obtained. In addition, there have been many anti-doping rule violations (ADRVs), both of which allow for relatively suitable analyses [6, 7]. All of the data used in the current study were obtained from the publicly available dataset provided by the IWF website, and was granted an exemption by the Institutional Review Board of Yonsei University. The acquired data were filtered and grouped for statistical analyses. A flow chart illustrating the details of the experimental design is shown in **Fig 1.**

### Data sampling and analyses

Publicly available data were obtained from the website of the International Weightlifting Federation (www.iwf.net) [8]. The results of all competitions organized by the IWF from 1998 to 2020 for men were collected. Data on weightlifters who had received a sanction for an anti-doping rule violation (ADRV) were obtained from the IWF Sanction List [9]. The total weight lifted was calculated as the sum of the best snatch and clean and jerk weights in which three chances were given for each. Athletes with missing performances or abnormal information were removed. Since athletes must be at least 15 years old to qualify for senior competitions, athletes under 15 were excluded. Athletes over 50 were also excluded because data showed that they were not distributed in most body weight categories. Moreover, we excluded the data if there was no valid record of three opportunities for both lifts. Finally, we acquired 27,029 datasets, 26,740 of which were the records of athletes with no history of sanctions (not-sanctioned group) and 289 of which were the records of athletes with a history of sanctions (sanctioned group). All performance records in the datasets used in this study, which included international competitions from 1998 to 2020, were treated as independent measurements. Age was classified into seven groups (15–19, 20–24, 25–29, 30–34, 35–39, 40–44, and 45–50). Body weight was classified into seven categories (61, 67, 73, 81, 96, 109, and +109) according to the 2020 Tokyo Olympics standards [10]. The number of datasets obtained for the sanctioned and not-sanctioned groups across age and body weight is presented in **Table 1**.

**Table 1:** Number of datasets obtained for the study.

Age (year)	Body weight category Sanction status	61	67	73	81	96	109	+109	61-+109
15–19	not-sanctioned	2405	1545	1672	1593	2277	679	684	10855
	sanctioned	8	12	12	13	12	5	5	67
20–24	not-sanctioned	986	1100	1363	1433	2570	1078	829	9359
	sanctioned	5	13	9	16	48	11	11	113
25–29	not-sanctioned	445	456	567	699	1304	730	532	4733
	sanctioned	5	7	13	3	34	14	10	86
30–34	not-sanctioned	123	154	158	192	371	245	252	1495
	sanctioned	1	3	2	1	4	3	4	18
35–39	not-sanctioned	18	17	26	40	74	55	30	260
	sanctioned	0	0	0	0	1	2	1	4
40–44	not-sanctioned	6	3	5	3	3	6	8	34
	sanctioned	0	0	0	1	0	0	0	1
45–50	not-sanctioned	0	0	1	2	0	0	1	4
	sanctioned	0	0	0	0	0	0	0	0
15–50	not-sanctioned	3983	3275	3792	3961	6600	2793	2336	26740
	sanctioned	19	35	36	34	99	35	31	289

### Statistical analyses

With the acquired data on weightlifting performance and ADRVs, various analyses of the relationship between athletic performance and doping were conducted. The significance of differences among mean values in total weight lifted was determined by one-way ANOVA with repeated measures, followed by the Bonferroni *post-hoc* test. Statistical significance was determined at an alpha level of 0.05 for all analyses. Data analyses were conducted using Python software (ver. 3.9.6; Python Software Foundation, Beaverton, OR, USA) to set up, steer, and analyze data results. Statistical research analysis was designed and carried out by configuring the modules in the Python package to check results according to the research progress.

## Results

### Weightlifting performance and sanction status across age

**Fig 2:**
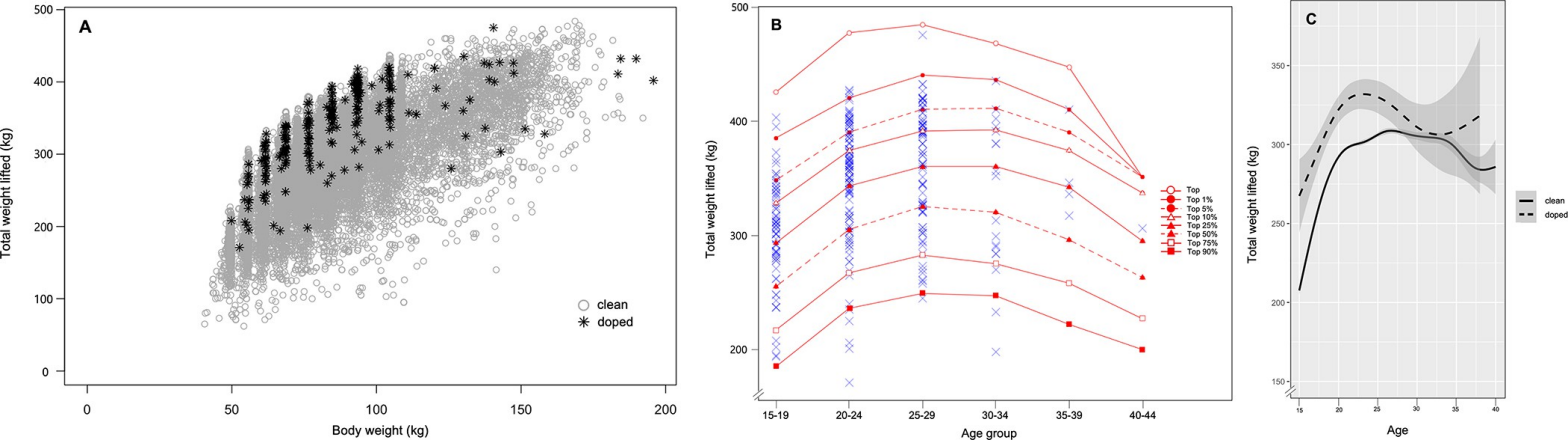
Male weightlifters’ performance in total weight lifted (kg) across age (26,740 results of not-sanctioned, 289 results of sanctioned). (A) Scatter plot showing the overall performance distribution for all athletes. *Gray open circles* represent results of not-sanctioned athletes, *and black asterisks* represent results of sanctioned athletes. (B) Age group analysis of performance (total weight lifted, kg) by age group for male weightlifters. *Solid line with open circles* represents performance of the top athlete, *solid line with filled circles* represents athletes in the top 1%, *dashed line with filled circles* represents athletes in the top 5%, *solid line with open triangles* represents athletes in the top 10%, *solid line with filled triangles* represents athletes in the top 25%, *dashed line with filled triangles* represents athletes in the top 50%, *solid line with open squares* represents athletes in the top 75%, and *solid line with filled squares* represents athletes in the top 90%. *Blue X’s* represent results of sanctioned athletes. (C) Line graph showing the trend of performance change. *Solid line* represents the mean of not-sanctioned athletes and *dashed line* represents the mean of sanctioned athletes, and shaded area indicates standard error.

In order to examine the overall distribution, age group analysis, and trend between weightlifting performance and sanction status across age, the results are presented as a scatter plot (**Fig 2A**) and line graphs (**Fig 2B** & **2C**), respectively.

From the overall distribution of male weightlifters’ performance across age (**Fig 2A**), many sanctioned cases were detected among athletes who performed better than the overall mean and those between their late teens and twenties. To determine the performance range in which sanctioned cases were present, performance level analysis using athletes’ performance pooled into the top 1%, 5%, 10%, 25%, 50%, 75%, and 90% for each age group was conducted, as shown in **Fig 2B**. Out of 289 sanctioned cases, 2.1% (6 cases), 14.5% (42 cases), and 14.5% (42 cases) were found among athletes who performed within the top 1%, top 1–5%, and top 5–10% of their performances, respectively. 29.1% (84 cases) and 18.7% (54 cases) were found within the top 10–25% and top 25–50% of their performances, respectively. Additionally, 13.9% (40 cases), 4.5% (13 cases), and 2.8% (8 cases) were found within the top 50–75%, top 75–90%, and top 90–100% of their performances, respectively. As shown in **Fig 2C**, there were significant differences between not-sanctioned and sanctioned athletes, and the difference was the greatest in their late thirties. The mean differences in performance between not-sanctioned and sanctioned athletes were 18.8% (not-sanctioned 254.9kg vs. sanctioned 302.8kg) in late teens, 15.4% (not-sanctioned 302.5kg vs. sanctioned 349.0 kg) in early twenties, 13.6% (not-sanctioned 317.5kg vs. sanctioned 360.8 kg) in late twenties, 6.0% (not-sanctioned 312.6kg vs. sanctioned 331.3kg) in early thirties, 20.6% (not-sanctioned 292.0kg vs. sanctioned 352.3kg) in late thirties, and 14.3% (not-sanctioned 267.8kg vs. sanctioned 306.0kg) in early forties. A more profound increase in performance was observed in male weightlifters between ages 15 to 25 (not-sanctioned 18.7% and sanctioned 15.2%). For the not-sanctioned group, performance continued to rapidly increase up to their early twenties, peaking at around 26 years of age, before falling from their thirties onward. For the sanctioned group, a similar trend was seen up to their early twenties, but instead of their performance falling like the not-sanctioned group, it increased in their thirties after the initial decline following peak performance. In the not-sanctioned group, there was an overall positive correlation between the athletes’ age and their athletic performance (r = 0.38, *p* < 0.05). The sanctioned group also showed an overall positive correlation between age and athletic performance (r = 0.26, *p* < 0.05).

### Weightlifting performance and sanction status across body weight

**Fig 3:**
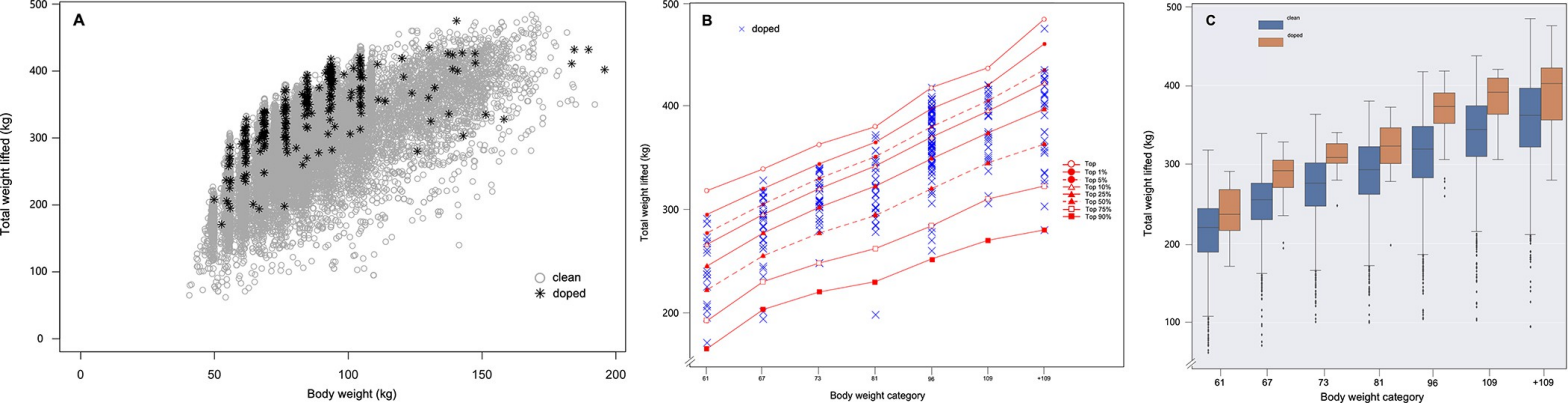
Male weightlifters’ performance in total weight lifted (kg) across body weight (26,740 results of not-sanctioned, 289 results of sanctioned). (A) Scatter plot showing the overall performance distribution for all athletes. *Gray open circles* represent results of not-sanctioned athletes, *and black asterisks* represent results of sanctioned athletes. (B) Body weight category analysis of performance (total weight lifted, kg) for male weightlifters. *Solid line with open circles* represents performance of the top athlete, *solid line with filled circles* represents athletes in the top 1%, *dashed line with filled circles* represents athletes in the top 5%, *solid line with open triangles* represents athletes in the top 10%, *solid line with filled triangles* represents athletes in the top 25%, *dashed line with filled triangles* represents athletes in the top 50%, *solid line with open squares* represents athletes in the top 75%, and *solid line with filled squares* represents athletes in the top 90%. *Blue X’s* represent results of sanctioned athletes. (C) Box plots of the average performance and outliers of each bodyweight category grouped by sanction status (not-sanctioned or sanctioned) for comparison. Boxes indicate the 25^th^ and 75^th^ percentiles. Whiskers indicate 10^th^ and 90^th^ percentiles with the middle horizontal line representing the mean. The outliers are indicated by dots.

A scatter plot, line graph, and boxplot were used to demonstrate the overall distribution (**Fig 3A**), the body weight category analysis (**Fig 3B**), and statistics (**Fig 3C**) of weightlifting performance based on sanction status across body weight, respectively.

The overall distribution of male weightlifters’ performance across body weight (**Fig 3A**) shows that many sanctioned cases were detected among athletes who performed better than the overall average, including more sanctioned athletes who weighed in exactly at or close to the cut-off weight for each body weight category. To determine the performance range in which sanctioned cases were present, performance level analysis using athletes’ performance pooled into the top 1%, 5%, 10%, 25%, 50%, 75%, and 90% for each body weight category was conducted, as shown in **Fig 3B**. Out of 289 sanctioned cases, 5.9% (17 cases), 19.0% (55 cases), and 15.2% (44 cases) were found among athletes who performed within the top 1%, top 1–5%, and top 5–10% of their performances, respectively. 26.0% (75 cases) and 20.4% (59 cases) were found within the top 10–25% and top 25–50% of their performances, respectively. Additionally, 9.0% (26 cases), 3.1% (9 cases), and 1.4% (4 cases) were found within the top 50–75%, top 75–90%, and top 90–100% of their performances, respectively. From the mean weightlifting performance of each bodyweight category (**Fig 3C**), there were significant differences between not-sanctioned and sanctioned athletes, and the difference was the greatest in the body weight category of 96kg. The mean differences in performance between not-sanctioned and sanctioned groups were 12.2% (not-sanctioned 214.9kg vs. sanctioned 241.2kg) in the 61kg weight category, 13.5% (not-sanctioned 249.8kg vs. sanctioned 283.4 kg) in the 67kg weight category, 14.4% (not-sanctioned 207.4kg vs. sanctioned 309.2 kg) in the 73kg weight category, 11.6% (not-sanctioned 287.4kg vs. sanctioned 320.9kg) in the 81kg weight category, 18.6% (not-sanctioned 310.1kg vs. sanctioned 367.8kg) in the 96kg weight category, 14.7% (not-sanctioned 334.1kg vs. sanctioned 383.2kg) in the 109kg, and 9.6% (not-sanctioned 353.7kg vs. sanctioned 387.6kg) in the +109kg weight category. Overall, there was a strong positive correlation between body weight and weightlifting performance in both the not-sanctioned group (r = 0.62, *p* < 0.05) and the sanctioned group (r = 0.68, *p* < 0.05).

### Weightlifting performance and sanction status across age and body weight

**Fig 4:**
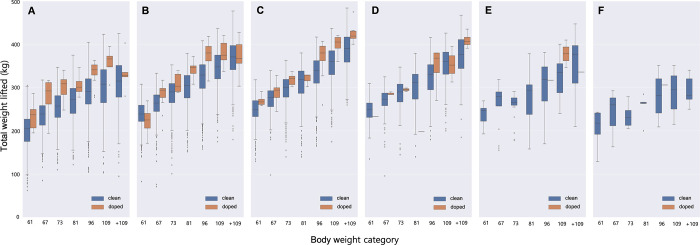
Male weightlifters’ performance in total weight lifted (kg) for each body weight category in the ages of 15–19 (A), 20–24 (B), 25–29 (C), 30–34 (D), 35–39 (E), and 40–44 (F) (26,740 results of not-sanctioned, 289 results of sanctioned). Box plots of the average performance and outliers of each body weight category are grouped by sanction status (not-sanctioned or sanctioned) for comparison. Boxes indicate the 25^th^ and 75^th^ percentiles. Whiskers indicate 10^th^ and 90^th^ percentiles, with the middle horizontal line representing the mean. Dots indicate the outliers.

In order to determine the typical range of weightlifting performance of sanctioned athletes for each body weight category in the different age groups, the results were presented as boxplots (**Fig 4**).

In the 15–19 yrs age group, the performance ranges of sanctioned athletes were 195-286kg for the body weight category of 61kg, 194-316kg for 67kg, 248-340kg for 73kg, 278-346kg for 81kg, 278-363kg for 96kg, 306-395kg for 109kg, and 280-403kg for +109kg (**Fig 4A**). In the 20–24 yrs age group, the performance ranges of sanctioned athletes were 171-269kg for the body weight category of 61kg, 201-317kg for 67kg, 288-339kg for 73kg, 308-372kg for 81kg, 315-418kg for 96kg, 337-420kg for 109kg, and 303-427kg for +109kg (**Fig 4B**). In the 25–29 yrs age group, the performance ranges of sanctioned athletes were 258-291kg for the body weight category of 61kg, 245-328kg for 67kg, 302-337kg for 73kg, 302-329kg for 81kg, 260-407kg for 96kg, 360-420kg for 109kg, and 400-475kg for +109kg (**Fig 4C**). In the 30–34 yrs age group, the performance ranges of sanctioned athletes were 284-290kg for the body weight category of 67kg, 291-299kg for 73kg, 270-380kg for 96kg, 313-395kg for 109kg, and 391-435kg for +109kg. There was only one record of a sanctioned athlete in each of the 61kg and 81kg weight categories (**Fig 4D**). In the 35–39 yrs age group, the performance range of sanctioned athletes was 346-41kg for the body weight category of 109kg. There was only one record of a sanctioned athlete in each of the 96kg and +109kg weight categories, and no sanctioned athletes in the body weight categories of 61kg, 67kg, 73kg, and 81kg (**Fig 4E**). In the 40–44 yrs age group, with the exception of one record, there were no sanctioned cases. The mean differences in performance between not-sanctioned and sanctioned groups across both age and body weight was the largest in the 15–19 yrs age group. In the 15–19 yrs age group, the mean differences in performance between not-sanctioned and sanctioned groups were 17.2% (not-sanctioned 201.4kg vs. sanctioned 236.0kg) for the body weight category of 61kg, 20.0% (not-sanctioned 234.6kg vs. sanctioned 281.5kg) for 67kg, 18.4% (not-sanctioned 253.9 vs. sanctioned 300.8kg) for 73kg, 13.3% (not-sanctioned 269.0kg vs. sanctioned 304.8kg) for 81kg, 15.6% (not-sanctioned 288.0kg vs. sanctioned 332.8kg) for 96kg, 18.8% (not-sanctioned 301.0kg vs. sanctioned 357.5kg) for 109kg, and 7.4% (not-sanctioned 311.1kg vs. sanctioned 334.2kg) for +109kg.

## Discussions

Based on Code 4.5.3.b of WADA’s international standard, an athlete’s performance history, including patterns and changes in their performance, can be used to determine which athletes should be targeted for doping testing [4]. We examined the potential roles of applying athletic performance analyses to improve anti-doping measures by comparing the performance of not-sanctioned and sanctioned groups across age and body weight. The data used for analyses consisted of the performance records and sanction status of male weightlifters from all international competitions, which were obtained from the IWF website. While prior analyses have been conducted using performance data in the different disciplines of athletics [11, 12], this study aimed to facilitate the development of a more scientific and systematic anti-doping program that aligns with the international standards supported by prior studies that analyzed running performance. We pooled athletic performance and sanction status from male weightlifters to conduct comparative analyses. Our results demonstrated an effect of sanctioned athletes’ performance relative to their age and body weight, and ultimately provided evidence that supports the implementation of the APP in possible doping.

For male weightlifters, the performance difference between the not-sanctioned and sanctioned groups was the greatest in their late thirties and the 96kg body weight category. A particularly rapid performance improvement was observed in ages 15–25, which is in line with the previous findings by Solberg et al. (2019) that analyzed peak performance in weightlifting [13] where a sharp increase in performance was observed for male athletes in their late teens and early twenties. Solberg et al. (2019) demonstrated that 2–3% of male athletes’ annual performance improvement occurs during this time frame. The reason for the most significant changes in performance being in the late thirties is unclear. However, since it was demonstrated that athletes’ performance generally declines in their thirties [14], doping can be inferred to have attenuated or inhibited this decline, allowing the sanctioned group to perform much better than the not-sanctioned group.

The difference in performance between athletes who were and were not sanctioned was greatest in the 96kg body weight category. However, since the datasets used in this study do not guarantee that the previous records of sanctioned athletes were clean performances, one instance of getting caught for doping only indicates that the sanctioned athlete was caught at that particular event while leaving open the possibility of prior doping violations that were not detected or tested for. In this study, sanctioned athletes’ previous records were analyzed as being clean, when in fact, those athletes may have been doping long before, thereby creating a wider disparity between the two groups, such as that in the 96kg body weight category, as shown in **Table 1**.

For the male weightlifters in this study, it is apparent that peak performance occurred in their twenties, and the trend of their changes in performance after the peak differed depending on their sanction status. Additionally, the performance of the sanctioned group being distributed in the high ranks was more evident when weightlifting performance and sanction status were analyzed across body weight, compared to when analyzed across age. However, more research is needed to explain the underlying difference between not-sanctioned and sanctioned groups.

Currently, random doping tests are enforced at times, but most doping tests are conducted on famous athletes or upper-ranked athletes. However, **Figs 2B** and **3B** indicate that doping is caught, not only among top-class athletes, but also among athletes who perform below the top 50% and even below the top 90%. Therefore, the APP should consider adopting the standards of the ABP that are not based on absolute standards, but on each athlete’s performance changes to develop a better targeting strategy [15]. More specifically, since the results of the age and body weight category analysis determined that sanctioned cases were most prevalent among athletes who performed in the top 10–25%, it is suggested that more athletes in this performance range should be selected as candidates for testing.

**Fig 4** presents the typical ranges of weightlifting performance of sanctioned athletes for each age group and body weight category. The efficiency of target testing can be enhanced by selectively analyzing the performance profiles of athletes whose performance falls within these ranges for their age group and corresponding body weight category. In light of the importance of anti-doping, performance analyses can be employed to increase the efficiency of doping detection by improving the targeting of suspected athletes for testing. Furthermore, when considering the importance of anti-doping in protecting the health of athletes and maintaining fairness in sports, some doping tests need to be targeted at athletes with poor athletic performance.

The results of the present study demonstrate that a marked increase in athletic performance outside of an athlete’s typical range is a reliable indicator of the need for further investigation. An advantage of athletic performance analyses is its use in establishing an inspection allocation plan. By understanding the relationship between performance changes and ADRVs, violations can be better detected, allowing more attention to be focused on testing athletes of events, age groups, and weight categories found to have larger changes in performance. Using both the ABP and APP, targeting athletes for testing can be more efficient, and recurring issues such as the fairness of doping tests [16] and human rights violations [17] can be addressed. Accordingly, the implementation of both systems can decrease the budget needed for doping tests and ultimately play a significant role in the prevention of doping in sports [18, 19].

Currently, there is the ethical concern that the APP can incorrectly target athletes who have achieved performance improvement through acquired efforts [20]. However, continued research can strengthen the APP system to mitigate such issues. Through the comprehensive and specific application of sports science, our innate physiological limitations as humans can be considered to establish a more concrete range of athletic performance improvement resulting from acquired efforts. This can be a turning point for overcoming the APP’s current limitations and building the foundation for a more advanced and scientifically supported anti-doping system.

In order to expand the use of the method outlined in the present study, as well as other advanced methods, high-quality raw data on performance and doping violation records need to be acquired for each sport. Through the scientific investigation of several sports, a solid framework for the APP can be established to expand its application. With sufficient access to data, the APP can be introduced as a powerful tool in the fight against doping across all sports. When the APP program becomes concrete, the efficiency of targeting athletes at high risk for doping will be improved, thereby resulting in a more developed intelligence-led anti-doping system. Furthermore, the findings of this study can initiate a paradigm shift from the detection to the prevention of doping. Future studies need to track relative performance changes for each individual athlete when target testing using artificial intelligence algorithms to accurately distinguish between clean and doped athletes. Studies on the APP may help improve fairness in targeting athletes with a higher probability of doping risk and alleviate the controversy around doping tests violating athletes’ human rights.

## Conclusion

The current study results support those of previous studies on the APP that demonstrate the relationship between athletes’ performance and the potential violation of anti-doping rules. In male weightlifters, these results are interpreted to mean that 1) sanctioned athletes were present in all ranges of performances regardless of age and body weight, 2) there were even higher rates of sanctioned athletes found in the top 10–25% of performances in each age group and body weight category, 3) there were significant differences in performance between not-sanctioned and sanctioned groups for all body weight categories, excluding +109kg, in the ages of 15–19 and 20–24, 4) therefore, performance data can be effectively used to better target suspected athletes for doping testing. With further research on this issue, there is much potential for developing an intelligence-led approach for better targeting athletes at high risk for doping, thereby further strengthening the effectiveness of doping tests and existing anti-doping systems.

## Supporting information

S1 Dataset (XLSX)
